# Understanding Equity, Diversity, and Inclusion Within Canadian Radiation Oncology Training Programs: A National Survey of Residents and Fellows

**DOI:** 10.3390/curroncol32110623

**Published:** 2025-11-06

**Authors:** Stefan Allen, Amanda Farah Khan, Jolie Ringash, David Bowes, Reshma Jagsi, Zhihui Amy Liu, Glen Bandiera, Ian J. Gerard, Shaun K. Loewen, Jennifer Croke

**Affiliations:** 1 Department of Radiation Oncology, University of Toronto, Toronto, ON M5G 2M9, Canada; stefanc.allen@nshealth.ca (S.A.); jolie.ringash@uhn.ca (J.R.); 2 Department of Oncology, University of Calgary, Calgary, AB T2N 1N4, Canada; amanda.khan@albertahealthservices.ca (A.F.K.); shaun.loewen@albertahealthservices.ca (S.K.L.); 3 Department of Radiation Oncology, Dalhousie University, Halifax, NS B3H 4R2, Canada; david.bowes@nshealth.ca; 4 Department of Radiation Oncology, Emory University School of Medicine, Atlanta, GA 30322, USA; rjagsi@emory.edu; 5 Dalla Lana School of Public Health, University of Toronto, Toronto, ON M5S 1A1, Canada; zhihuiamy.liu@uhn.ca; 6 Department of Medicine, University of Toronto, Toronto, ON M5S 1A1, Canada; glen.bandiera@utoronto.ca; 7 Royal College of Physicians and Surgeons of Canada, Ottawa, ON K1S 5N8, Canada; 8 Division of Radiation Oncology, McGill University, Montreal, QC H3A 0G4, Canada; ian.gerard@mail.mcgill.ca

**Keywords:** diversity, equity, discrimination, radiation oncology, postgraduate medical education

## Abstract

**Simple Summary:**

This study characterizes the current representation of sociodemographic groups within Canadian radiation oncology training programs and trainees’ lived experiences. Most (83%) trainees reported training program satisfaction; however, at least one episode of discrimination during training was reported by 38%, mostly due to gender (16%) and race (15%). Women were more likely to feel under-represented in their program (46% vs. 13%, *p* = 0.001) and perceived more discrimination events (64% vs. 19%, *p* < 0.001). Areas for improvement identified from thematic analysis include EDI education, improved pathways for mistreatment reporting, and ensuring diverse selection committees.

**Abstract:**

Background: This study characterizes the current representation of sociodemographic groups within Canadian radiation oncology training programs and trainees’ lived experiences. Methods: A 59-item ethics-approved, bilingual survey assessed sociodemographics, training perceptions, mentorship, discrimination/harassment experienced, and open-ended questions. Electronic surveys were distributed to all Canadian radiation oncology residents/fellows. Descriptive statistics summarized survey responses. Categorical groups were compared using chi-squared/Fisher’s exact tests. Thematic analysis was performed on open-ended responses. Results: Between July and December 2023, 98 of 177 (56%) trainees participated: 70% were residents, 52% identified as male, 62% as a racialized minority, and 10% as a sexual minority. Most respondents reported training program satisfaction (83%) and a respectful workplace culture (69%); however, discrimination during training was reported by 38%. Less than half (45%) felt comfortable reporting discrimination/harassment within their workplace. Women were more likely to feel under-represented in-training (46% vs. 13%, *p* = 0.001) and perceived more discrimination events (64% vs. 19%, *p* < 0.001). Three themes emerged as follows: importance of offering EDI education, ensuring pathways for reporting learner mistreatment, and creating appropriately diverse selection committees. Conclusions: Although most Canadian radiation oncology trainees reported satisfaction and a respectful culture, key differences between groups were observed. Targeted strategies and stronger institutional policies to improve representation and reduce rates of discrimination/harassment are needed.

## 1. Introduction

Equity, diversity, and inclusion principles (EDI) have become embedded within strategic priorities in postgraduate medicine, academic medical institutions, and professional organizations [1,2,3,4,5,6]. Promoting a diverse workforce in healthcare has the potential to enhance cultural competency, reduce health disparities, and improve patient outcomes [7]. Furthermore, it is important that the healthcare workforce represents the population it serves. Within the United States (US) radiation oncology (RO) workforce, the proportion of women and under-represented minority groups is lower than that of the general population [8]. Studies have shown that cancer patient outcomes are worse for under-represented populations; therefore, efforts to promote diversity within medical schools and training programs have been suggested to improve patient care [9,10].

The American Society for Radiation Oncology (ASTRO) has established a Health Equity, Diversity, and Inclusion (HEDI) Council and the American Society of Clinical Oncology (ASCO) has established priorities for diversification as a mechanism for addressing health disparities and improving cancer care and research [1,2]. Additionally, within postgraduate medicine, the Association of Residents in Radiation Oncology (ARRO) established an Equity and Inclusion Subcommittee (EISC) in 2020 with the aim of creating a safe space for all trainees, promoting diversity, equity, inclusion, and social justice, and assessing diversity in the workforce [6]. Furthermore, the Canadian Association of Radiation Oncology (CARO) established an Equity, Diversity, Inclusion, and Indigenous Care Standing Committee in 2023 to promote awareness and support initiatives that improve diversification within the specialty to ensure the provision of high-quality, inclusive patient care [11].

Previously, most RO EDI research has been conducted in the US and has focused primarily on gender, specifically the underrepresentation of women [12,13]. However, more comprehensive studies evaluating and exploring EDI within RO and medical physics have been recently conducted. The European Society for Radiotherapy and Oncology (ESTRO) conducted a collaborative study to collect benchmark data on EDI and workforce engagement [14]. They found overall lower inclusion scores compared to US data and concluded that initiatives to improve EDI and engagement are needed. However, of the 812 respondents, only 12.6% were in-training, and unfortunately, their data were not reported separately. Furthermore, within the field of medical physics, the Canadian Organization of Medical Physics (COMP) in collaboration with the American Association of Physicists in Medicine (AAPM) recently published their faculty climate survey results [15,16]. Although most respondents reported positive workplace experiences, they identified equity-lacking groups, including women, racialized minorities, Indigenous peoples, and people with disabilities. Recommendations to improve EDI and accessibility included conducting routine EDI climate surveys, creating EDI training opportunities for society members, and conducting an EDI climate survey for trainees [15,16].

EDI data regarding RO trainees are emerging. In 2022, the ARRO EISC surveyed RO residents regarding diversity, equity, inclusion, and belonging. With a 42% response rate, they found significant racial, ethics, and gender differences in the areas of support, mentorship, inclusion, and bias [17]. They recommended that RO training programs collaborate to focus on initiatives directed toward residents from under-represented groups to foster a supportive and unbiased learning environment, which, in turn, should facilitate recruitment and retention of a diverse workforce in the future.

There are limited data regarding the demographics and experiences of RO trainees worldwide, and Canadian-specific data are lacking. The objectives of this study were to understand the sociodemographic and lived experiences of RO trainees in Canada to bring awareness to any gaps that exist and propose EDI strategies to improve representation within the specialty to enhance clinical care.

## 2. Materials and Methods

### 2.1. Study Population and Survey Measures

Following a comprehensive literature review of other published EDI surveys and international input from content and methodological experts, two questionnaires were created in tandem to collect cross-sectional data for Canadian radiation oncology trainees and faculty [14,15,16].

Prior to distribution, the surveys underwent cognitive pre-testing and pilot testing, whereby each survey item was reviewed for clarity and appropriateness. Subsequent iterative revisions were made when deemed necessary by the study team until consensus was reached on the final complete survey [18]. The final trainee survey included the following 59 items: sociodemographics (n = 24), training perceptions (n = 5), mentorship (n = 8), experiences of discrimination/harassment (n = 19), and open-ended questions (n = 3). Bilingual surveys were created to encourage survey participation in either of Canada’s official languages (Supplementary Materials). A description and results of the faculty survey are the topic of a separate manuscript [19].

The survey was electronically distributed via RedCAP between July 2023 and December 2023 [20]. The study population consisted of RO residents and fellows currently enrolled in a Canadian training program. Residency training in Canada is five years (PGY 1–5), and applicants apply through the Canadian Resident Matching Service (CaRMS). Fellows are trainees specializing in a particular field after completing am RO residency. The initial invite and three email reminders were sent to all RO trainees (n = 173) through Program Directors and to RO trainee members of CARO via their email registry. This study was approved by our local research ethics board (REB 19-5845.7).

### 2.2. Data Analysis

Descriptive statistics summarized responses. Comparisons between demographic groups (gender, race, level of training, location of training) were performed using chi-square and Fisher’s exact tests. Univariable and multivariate logistic regression analyses were performed to identify factors associated with the outcome of considering transferring to a different training program. Inductive thematic analysis was performed on open-ended items [21]. Reflexivity was acknowledged. Recurring responses were grouped into categories, which yielded emergent themes to encapsulate key findings. Themes were reviewed by collaborators for consensus and approval [22].

## 3. Results

### 3.1. Survey Response

Using data from the 2023–2024 Canadian Post-MD Education Registry (CAPER), we identified 115 residents and 58 fellows (173 trainees) enrolled in Canadian RO training programs [23]. Among these, 98 responded to the survey (57% response rate).

### 3.2. Respondent Demographics

Complete self-reported respondent demographics are highlighted in Table 1. Regarding level of training, 29% (28/98) were fellows and 65% (64/98) were residents with all postgraduate years represented. Of the resident respondents, most were Canadian Medical Graduates (CMGs) (61/64, 95%), whereas most fellow respondents were International Medical Graduates (IMGs) (24/28, 86%). Regarding gender identity, 52% (51/98) identified as men, 42% (41/98) as women, and 3% (3/98) as non-binary. Gender distribution according to the level of training was similar (*p* = 0.42). Twenty-eight respondents (28/98, 29%) reported that their primary language differed from English/French. Most respondents (61/98, 62%) reported that both of their parents/guardians had a college/university degree.

Most respondents (61/98, 62%) identified as a racialized minority, with the most common self-reported ethnicities being Chinese (16/98, 16%), South Asian (15/98, 15%), and Arab (10/98, 10%). Gender distribution in Caucasian and racialized groups was similar (*p* = 0.57). Fellow respondents were more likely to self-identify as a racialized minority compared to resident respondents (82% vs. 53%, *p* = 0.016). Regarding sexual orientation, 10% (10/98) identified as a sexual minority (2SLGBTIA+) [24]. When trainees were asked if they had a disability, 4% (4/98) responded “yes”, with a mental health/emotional disability being the most common.

Having a career with strong research or education components was desired/strongly desired by 31% (28/98) and 87% (79/98) of respondents, respectively. In addition to having a medical degree, 32% (31/98) reported having a Masters/PhD/equivalent, with 14% (13/98) currently pursuing an additional postgraduate degree. Half of the respondents (50%) reported being authors of 1–5 peer-reviewed publications, and 6% (5/98) reported being authors of more than 25 publications.

Respondent demographics were compared to CAPER and Canadian census data, when available. Our study population was representative of CAPER data with respect to the level of training (e.g., resident vs. fellow; *p* = 0.80) and representative of CAPER and Canadian census data with respect to gender identity (*p* = 0.24 compared with CAPER and *p* = 0.39 compared with census). Racialized groups were over-represented in our study compared with Canadian census data (62% vs. 27%, *p* < 0.001). This remained true when residents were analyzed separately (53% vs. 27%, *p* < 0.001). Black respondents were representative of the Canadian population (3% vs. 4.3%, *p* = 0.55); however, Indigenous respondents were under-represented (1% vs. 4.9%, *p* = 0.007).

### 3.3. Training Perceptions and Mentorship

Table 2 summarizes responses regarding training perceptions and mentorship. The majority reported program satisfaction (83%, 78/94) and a “culture of respect” (69%, 64/93). However, 40% (37/92) reported they have considered switching to a different training program at least once and 74% (69/93) reported feeling regret about deciding to become a physician at least once. When asked if “People like myself are under-represented in my training program”, 28% (25/90) responded “yes”, and 15% (13/88) responded “yes” to “There are equity and diversity obstacles for entry in my radiation oncology training program.”

Most respondents (58/91, 64%) reported that a formal mentorship program existed within their training program, with 73% (66/91) reporting having at least one faculty mentor and 81% (74/91) turning to peers for mentorship. However, 21% (19/91) reported it was difficult/very difficult to identify a mentor. The most common topics discussed with a mentor included research (53%), clinical work (52%), and career planning (43%). As residents progressed in their training, they reported increased mentorship discussions in career planning (*p* = 0.005) and fellowships (*p* = 0.003). Most respondents (67/91, 75%) were satisfied/very satisfied with their current mentorship and 40% (36/91) agreed/strongly agreed with the statement, “It is important I have a mentor with similar demographic characteristics to me.”

Univariable logistic regression analysis was used to identify factors associated with considering transferring to a different training program and included dissatisfaction with current mentorship (OR = 17.14, *p* = 0.002), lack of comfort reporting harassment incidents (OR = 10.56, *p* = 0.001), dissatisfaction with their training program (OR = 5.77, *p* = 0.034), perceived equity and diversity obstacles for entry into an RO training program (OR = 4.15, *p* = 0.028), personal experience with discrimination (OR = 3.05, *p* = 0.025), and personal experience with harassment (OR = 2.90, *p* = 0.049) (Table 3).

### 3.4. Discrimination and Harassment

Table 4 summarizes experiences of discrimination and harassment. At least one episode of discrimination during training was reported by 38% of respondents (28/75), primarily due to gender (16%), race (15%), and age (10%). At least one episode of personal harassment was reported by 24% and was mainly perpetrated by a patient/family member (56%, 9/16), followed by a faculty member (8/16, 50%). Less than half of respondents (45%) felt comfortable reporting discrimination and/or harassment, and only 11% reported disclosing harassment/discrimination experiences.

Training to address sexual harassment, anti-racism, LGBTQ health, and other forms of discrimination was available to 47%, 53%, 33%, and 50% of respondents, respectively. Up to 49% of respondents were not aware if these types of training existed.

### 3.5. Comparisons by Demographic Groups

Women were more likely to feel under-represented in their training program (17/41, 46% vs. 6/51, 13%, *p* = 0.001) and to perceive equity and diversity obstacles (8/41, 22% vs. 2/51, 4%, *p* = 0.02). However, there were no differences between level of training (residents vs. fellows) (*p* = 0.43 and *p* = 1.0), location of training (CMGs vs. IMGs) (*p* = 0.30 and *p* = 0.43), race/ethnicity (*p* = 0.81 and *p* = 1.0), or primary language (*p* = 0.87 and *p* = 0.38). Women were also more likely to strongly agree/agree to the statement “It is important I have a mentor with similar demographic characteristics to me” (21/41, 55% vs. 13/51, 28%, *p* = 0.03).

Women perceived more discrimination events (64% vs. 19%; *p* < 0.001), whereas male respondents felt more comfortable reporting harassment incidents (60% vs. 26%; *p* = 0.01). Women were also more likely to perceive discrimination based on gender (15/41, 37% vs. 1/51, 2%, *p* < 0.001) and age (8/41, 20% vs. 2/51, 4%, *p* = 0.02) compared to men. Harassment events were more commonly perceived by resident respondents compared to fellow respondents (16/64, 25% vs. 1/28, 4%, *p* = 0.018), by Caucasian respondents compared to racialized minorities (32% vs. 10%, *p* = 0.011), and by CMG respondents compared to IMGs (18/70, 26% vs. 0/28, 0%, *p* < 0.001). Racialized minorities were more likely to rate an “excellent/very good” culture of respect compared to Caucasian respondents (44/61, 77% vs. 20/37, 55%, *p* = 0.043), although they reported more discrimination due to race/ethnicity and national origin (25% vs. 0%; *p* < 0.001 and 11% vs. 0%, *p* = 0.043, respectively). Residents were more likely to feel discriminated against by patients/family due to gender compared to fellow respondents (14/64, 22% vs. 1/28, 4%, *p* = 0.03).

### 3.6. Thematic Analysis

Open-ended questions were included to further explore trainees’ experiences and to seek suggestions for improving the learning environment and advancing EDI. Questions included are as follows: “What should training programs do to address learner mistreatment?”, “What should training programs do to advance equity, diversity, and inclusion in the workplace?”, and “What should training programs do to make resident selection or faculty hiring practices more equitable?” There were 24 (24%), 20 (20%), and 21 (21%) free-text responses, respectively, to these questions.

Three themes were generated as follows (Table 5):

(1) *Importance of programs providing education and training to faculty on EDI and learner mistreatment*

Respondents recommended EDI be embedded within faculty development initiatives at the departmental level. Education on learner mistreatment was also recommended, including unconscious bias training. These recommendations were felt to advance EDI in the workplace and help address learner mistreatment.

(2) *Programs must establish best practices for trainee reporting of mistreatment*

Respondents highlighted the importance of universities, departments, and training programs working collaboratively to create clear and explicit pathways and procedures for reporting mistreatment that is confidential and anonymous. These strategies should increase resident comfort in reporting mistreatment and foster safe spaces.

(3) *Selection committee and processes need to acknowledge EDI*

Respondents believe that selection committees should consist of diverse representation, members should complete mandatory unconscious bias training, and conduct “blind” review of applicants using transparent and standardized evaluation metrics to mitigate bias. These strategies should improve equity in resident selection.

## 4. Discussion

This is the first national survey to characterize the current sociodemographics and learning environment experiences of Canadian RO trainees. Self-reported under-represented groups were identified, highlighting opportunities to improve diversity and inclusion. Although most respondents reported satisfaction and respectful cultures, key differences between groups were observed, including experiences with discrimination and harassment.

In our survey, women comprised 42% of respondents, which is representative of the Canadian RO trainee population and census data. Our results are comparable to US findings for RO residents (39.2%) but lower than the 59% of current medical students identifying as women in Canadian medical schools [25]. Additionally, we found no significant differences regarding female representation according to race/ethnicity or PGY level. Women more often perceived encountering equity and diversity obstacles in their training, reported more discrimination events, and felt less comfortable reporting harassment incidents compared to male respondents. Similarly, ARRO reported that women were less likely to agree that they were able to voice a contrary opinion without fear of retaliation compared to male residents [17]. This highlights that gender-based disparities continue to exist within RO. We recommend targeted mentorship programs and strategies to mitigate bias and increase representation of female medical students interested in RO.

Caucasian respondents were under-represented compared to Canadian census data (38% vs. 73%, *p* < 0.001), with most respondents (62%) identifying as racialized minorities. The most common self-reported race/ethnicities were Chinese, Southeast Asian, and Arab. Respondents from these groups were over-represented compared to Canadian census data, even when fellows, who were more commonly IMGs, were analyzed separately from resident respondents. In the ARRO survey, 54.6% identified as Caucasian (vs. 32% in our study), with Asian being the most common racialized group (29.5%) [17]. Indigenous respondents were under-represented (1% vs. 5%, *p* = 0.007). Efforts to improve the representation of Indigenous populations in medicine have been recognized as needed and include breaking down systemic barriers to make education more accessible, establishing strategies to support students longitudinally, and adopting a distinctions-based approach to recruitment and selection processes [26].

Similar to ARRO’s findings, most respondents reported a respectful workplace culture [17]. However, at least one episode of discrimination during training was reported by 38% of respondents, which is concerning and warrants further assessment. The top three perceived reasons for personal discrimination were gender, race/ethnicity, and age. Work performed within Medical Physics faculty showed that experiences of discrimination were primarily related to gender, age, and pregnancy/caretaking responsibilities [15,16]. In response, AAPM/COMP created recommendations to improve the workplace environment, including creating an online, non-punitive incident reporting system for discrimination/harassment [15,16]. Similar policies should also be implemented within RO training programs, as nearly half of respondents felt uncomfortable reporting discrimination/harassment experiences.

Thematic analysis provided insights into how training programs can address learner mistreatment, advance EDI, and improve equity in selection processes. Our themes are similar to recommendations conceived by ARRO and other residency programs [17,27]. Additionally, it is recommended that programs focus on developing inclusive cultures for psychological safety, establishing cross-institutional and cross-specialty collaborations, and creating mentorship opportunities for under-represented groups to increase workforce diversification through recruitment and outreach. In Canada, our professional organization CARO has implemented an Under-represented in Radiation Oncology Mentorship Program (UROMP) [28] to increase access to mentorship, foster inclusive environments, and help improve diversity within the specialty. Additionally, ESTRO piloted a multidisciplinary, international mentoring program to facilitate professional development [29]. The pilot was positively endorsed by participants and is now an annual program commencing at the ESTRO Congress. Furthermore, we also recommend that efforts be directed to undergraduate medical education, with initiatives focused on medical school diversification and removal of barriers to residency entry. For example, 67% of medical students interested in radiology reported that a diverse and inclusive learning environment impacted their program ranking selections [30]. In 2021, CaRMS established EDI as a strategic priority, with the goal of collecting diversity data to inform the current environment and future improvements in residency applications [28]. One way of accomplishing this is through a voluntary CaRMS self-identification questionnaire that allows applicants to confidentially report whether they identify as a member of an equity-deserving group [31]. These initiatives will lay the foundation to identify inequities, helping training programs address these gaps.

Our study has several limitations. First, there is a potential selection/response bias, as those with strong opinions (positive or negative) may have been more likely to respond. For example, Caucasian respondents were under-represented compared to Canadian census data. Race data are not collected via CAPER; however, our respondents were representative of the Canadian radiation oncology trainee population regarding gender identity and level of training. Second, some demographic groups were small (e.g., 2SLGBTQIA+ and Indigenous respondents), and given the low numbers, further analyses were not possible, limiting the conclusions that can be made. Third, no formal correction for the family-wise error rate was made, as the comparisons were largely exploratory and descriptive in nature; therefore, the reported *p*-values should be interpreted with caution, and findings may be subject to increased risk of Type I error.

## 5. Conclusions

We characterized the current EDI characteristics of sociodemographic groups within Canadian RO training programs and assessed trainees’ lived experiences. Although the majority reported satisfaction and a respectful culture within their programs, key differences between groups were observed, including experiences with discrimination and harassment. Targeted strategies and stronger institutional policies to improve representation and reduce rates of discrimination and harassment in Canada’s RO training programs are needed and recommended. Future work should include collaboration with CARO and RO training program leadership to formally implement EDI initiatives and establish priorities to promote, recruit, and retain diverse members while improving workplace environments and training programs for all.

## Figures and Tables

**Table 1:** Demographic data of survey respondents.

Demographic	Response N = 98	2022–2023 CAPER Data ^1^	2021 Census Data – Canadians ^2^	Significance/Notes
**Gender Identity** Man Woman Non-binary Gender fluid I do not know Prefer not to answer	51 (52%) 41 (42%) 1 (1%) 1 (1%) 1 (1%) 4 (4%)	79 (64.2%) 44 (35.8%)	51% 49%	*p* = 0.24 (compared to CAPER) *p* = 0.39 (compared to Census)
**Level of Training** Residents PGY1 PGY2 PGY3 PGY4 PGY5 Fellow Prefer not to answer Missing	64 (67%) 13 (14%) 14 (15%) 15 (16%) 16 (17%) 6 (6%) 28 (30%) 3 (3%) 3	119 (67%) 58 (33%)	-	*p* = 0.80 (compared to CAPER)
**Age** 25–34 35–44 ≥45	81 (83%) 14 (14%) 3 (3%)			
**Sexual Orientation** Heterosexual Gay Bisexual Asexual Queer Pansexual Questioning Prefer not to answer	81 (84%) 3 (3%) 2 (2%) 1 (<1%) 1 1 2 (2%) 7 (7%)			
**Race/Ethnicity** Caucasian/White Racialized Group Chinese South Asian Arab Filipino Black Indigenous Other	37 (38%) 61 (62%) 16 (16%) 15 (15%) 10 (10%) 7 (7%) 3 (3%) 1 (1%) 9 (9%)		73% 27% 4.7% 7.1% 1.9% 2.6% 4.3% 5.0%	*p* < 0.001 (compared to Census) *p* < 0.001 *p* < 0.001 *p* = 0.001 *p* = 0.005 *p* = 0.55 *p* = 0.07
**Race and Gender** **(subset analysis)** Caucasian overall Caucasian male Caucasian female Racialized overall Racialized male Racialized female	37 (38%) 19 (51%) 17 (46%) 61 (62%) 32 (52%) 24 (39%)			Gender distribution in Caucasian and racialized groups was similar, *p* = 0.57
**Race and Level of Training** **(subset analysis)** Residents overall Caucasian Racialized group Fellows Caucasian Racialized group Prefer not to answer Missing	64 (68%) 30 (47%) 34 (53%) 28 (30%) 5 (18%) 23 (82%) 3 (6%) 1			Level of training in Caucasian and Racialized groups was significantly different, *p* = 0.016
**Religion** Christianity Atheist/No religion Islam Other Prefer not to answer Missing	24 (25%) 41 (43%) 10 (10%) 12 (13%) 9 (9%) 2			
**Citizenship Status** Canadian By birth By immigration Permanent Resident Work Visa	70 (71.4%) 58 (59.2%) 12 (12.2%) 1 (1%) 27 (27.6%)		33.1 m (91%) 27 m (74%) 6.1 m (17%) - -	
**Marital Status** Married/Domestic Single Prefer not to answer	57 (58.2%) 39 (39.8%) 2 (2%)		4.9 m (57%) 3.7 m (43%)	
**Number of dependents** 0 1 2 3+	74 (75.5%) 10 (10.2%) 10 (10.2%) 3 (3.0%)			
**Primary language** English French Another language Prefer not to answer	58 (59%) 11 (11%) 28 (29%) 1 (1%)		27.8 m (76%) 8 m (22%) 0.67 m (2%)	
**Degrees earned** MD Masters PhD MBA	98 (100%) 26 (27%) 5 (5%) 3 (3%)			
**Residency Training Location** Canada Elsewhere Missing	64 (77%) 19 (23%) 15			
**Parents/guardians with college/university degree(s)** One Both Neither Prefer not to answer	25 (26%) 61 (62%) 11 (11%) 1 (1%)			
**Approximate household income as a teenager** $150,000 + $100,000–$150,000 $50,000–$100,000 <$50,000 I don’t know Prefer not to answer	26 (27%) 11 (11%) 27 (28%) 18 (18%) 13 (13%) 3 (3%)			
**Number of peer-reviewed publications** 0 <5 5–10 10–25 >25	12 (13%) 45 (50%) 17 (19%) 11 (12%) 5 (6%)			
**Do you view yourself as having a disability?** Yes No Prefer not to answer	4 (4%) 91 (93%) 3 (3%)		27% ^3^ 73% N/A	

^1^ Canadian Post-MD Education Registry (CAPER). Individual Specialty Reports. https://caper.ca/postgraduate-medical-education/individual-specialty-reports (accessed on 1 April 2025). ^2^ Statistics Canada—Data Tables for the 2021 Census of Population. Ottawa, ON, Canada, 2021. https://www12.statcan.gc.ca/census-recensement/2021/dp-pd/dt-td/index-eng.cfm. (accessed on 1 April 2025) ^3^ Statistics Canada—Data Tables for the 2022 Canadian Survey on Disability. Ottawa, ON, Canada, 2022. https://www23.statcan.gc.ca/imdb/p2SV.pl?Function=getSurvey&SDDS=3251. (accessed on 1 April 2025)

**Table 2:** Job perceptions and mentorship status of survey respondents.

Question		Study Sample
**All in all, I feel satisfied with my training program.**	Disagree/strongly disagree	8 (9%)
	Neither agree nor disagree	8 (9%)
	Agree/strongly agree	78 (83%)
	Missing	4
**How often have you considered transfer to a different training program?**	Never	55 (60%)
	Once or twice/sometimes	35 (38%)
	Often/many times	2 (2%)
	Missing	6
**How often have you felt regret about deciding to become a physician?**	Never	24 (26%)
	Once or twice/sometimes	57 (61%)
	Often/many times	12 (13%)
	Missing	5
**A formal mentorship program exists within my department.**	Yes	58 (64%)
	No	33 (64%)
	Missing	7
**How much do you turn to peers for mentorship?**	Never	2 (2%)
	Less than monthly	15 (16%)
	Monthly	23 (25%)
	Weekly	33 (36%)
	Daily	18 (20%)
	Missing	7
**I currently have at least one faculty mentor.**	Yes	66 (73%)
	No	25 (27%)
	Prefer not to answer	7
**It is important I have a mentor with similar demographic characteristics to me.**	Disagree/strongly disagree	31 (33%
	Neither agree nor disagree	24 (26%)
	Agree/strongly agree	36 (40%)
	Missing	7
**How easy has it been for you to identify someone whose career could serve as a model for your own?**	Easy/very easy	41 (45%)
	Neither easy nor difficult	31 (34%)
	Difficult/very difficult	19 (21%)
	Missing	7
**Overall, I am happy with the mentorship I currently receive.**	Dissatisfied/very dissatisfied	9 (10%)
	Neither satisfied nor dissatisfied	13 (15%)
	Satisfied/very satisfied	67 (75%)
	Missing	9
**People like me are under-represented in my training program.**	Yes	25 (28%)
	No	65 (72%)
	Missing	8
**There are equity and diversity obstacles for entry into my radiation oncology training program.**	Yes	13 (15%)
	No	75 (85%)
	Missing	10

**Table 3:** Factors associated with considering transferring to a different training program.

	OR(95%CI)	*p*-Value
**All in all, I am satisfied with my training program.**		**0.034**
Agree/strongly agree	Reference	
Neither agree nor disagree	3.21 (0.73, 16.63)	0.13
Disagree/strongly disagree	5.77 (1.23, 41.31)	**0.040**
**How often have you felt regret about deciding to become a physician?**		0.053
Never	Reference	
Sometimes/once or twice	2.09 (0.75, 6.50)	0.18
Many times/often	6.00 (1.39, 30.20)	**0.020**
**Overall, I am happy with the mentorship I currently receive.**		**0.002**
Very satisfied/somewhat satisfied	Reference	
Neither satisfied nor dissatisfied	2.50 (0.74, 8.67)	0.14
Very dissatisfied/somewhat dissatisfied	17.14 (2.88, 328.76)	**0.009**
**There are equity and diversity biases obstacles for entry into my radiation oncology training program**		**0.028**
No	Reference	
Yes	4.15 (1.23, 16.56)	
**How often if at all did you personally experience discrimination during your residency fellowship program?**		**0.025**
Never	Reference	
Once/sometimes/regularly	3.05 (1.16, 8.29)	
**How often did you personally experience harassment during the course of your training program?**		**0.049**
Never	Reference	
Once/sometimes/regularly	2.90 (1.02, 8.75)	
**Please rate your level of agreement with the following statement: I feel comfortable reporting harassment incidents at my workplace.**		**0.001**
Agree/strongly agree	Reference	
Neither agree nor disagree	2.17 (0.71, 6.87)	0.18
Disagree/strongly disagree	10.56 (2.88, 46.81)	**<0.001**

**Table 4:** Workplace culture, discrimination, and harassment experiences survey respondents.

Question		Study Sample
**Thinking about the past year, how would you rate the culture of respect in your department?**	Excellent	28 (30%)
	Very good	36 (39%)
	Good	15 (16%)
	Adequate	8 (9%)
	Poor	5 (5%)
	Very poor	1 (1%)
	Missing	5
**How often if at all did you personally experience discrimination in your residency/fellowship program?**	Never	47 (63%)
	Once	6 (8%)
	2–4 times	14 (19%)
	5–10 times	6 (8%)
	Regularly/ongoing basis	2 (3%)
	Missing	23
**During your residency/fellowship training on what basis have you felt discriminated upon?**	Gender	16 (16%)
	Race/ethnicity	15 (15%)
	Age	10 (10%)
	National origin	7 (7%)
	Childcare/caregiving	4 (4%)
	Other	13 (13%)
**Since the start of your training program, have you personally encountered harassment at your workplace?**	Yes	18 (18%)
	No	80 (82%)
**How often did you personally experience harassment during the course of your training program?**	Once	2 (11%)
	2–4 times	11 (61%)
	5–10 times	4 (22%)
	Regularly/ongoing basis	1 (6%)
	Missing	80
**I feel comfortable reporting harassment incidents at my workplace.**	Disagree/strongly disagree	17 (22%)
	Neither agree/disagree	25 (32%)
	Agree/strongly agree	35 (45%)
	Missing	21
**What was the role of the person(s) who harassed/discriminated against you?**	Faculty member	8 (50%)
	Patient/patient’s family	9 (56%)
	MD student/resident/fellow	5 (31%)
	Nurse	3 (19%)
	Administrator	1 (6%)
**Was the person who harassed/discriminated against you someone in a position to directly affect your academic, and/or professional opportunities?**	Yes	6 (6%)
	No	7 (7%)
	Not sure	4 (4%)
**Did you tell anyone about these experiences?**	Yes	11 (11%)
	No	5 (5%)
**If you experienced harassment/discrimination perpetrated by a patient/family, what was it based on?**	Gender	15 (15%)
	Age	10 (10%)
	Race/ethnicity	11 (11%)
	National origin	6 (6%)
	Other	5 (5%)
**Within your training program university is there training provided to address sexual harassment?**	Yes	36 (47%)
	No	13 (17%)
	Not sure	28 (36%)
	Missing	21
**Within your training program university is there training provided to address racism?**	Yes	41 (53%)
	No	15 (19%)
	Not sure	21 (27%)
	Missing	21
**Within your training program university is there training provided to address LGBTQ health?**	Yes	25 (33%)
	No	14 (18%)
	Not sure	37 (49%)
	Missing	22
**Within your training program university is there training provided to address other forms of discrimination?**	Yes	38 (50%)
	No	9 (12%)
	Not sure	29 (38%)
	Missing	22

**Table 5:** Thematic analysis and selected quotes from open-ended questions.

Theme	Selected Summarized and Representative Quotes
**Importance of programs providing education and training to faculty on EDI and learner mistreatment**	“Having workshops for equity and diversity organized by the department itself, not just the PGME.”
	“Hiring committees should have undergone training to mitigate bias.”
	“…establish diversity and inclusion policies, provide implicit bias/trauma-informed teaching training (mandatory—especially for leadership), and implement EDI recruitment/promotions practices (and incentives for this) …provide ongoing training and education in EDI best practices…”
**Programs must establish best practices for trainee reporting of mistreatment**	“Create more streamlined reporting systems and keep them anonymous. I have not reported some instances of mistreatment because given the small community I felt I would almost certainly be identified if giving critical feedback.”
	“Confidential avenues to report mistreatment, opportunities to debrief with supportive and trained faculty members, and cultivate an environment where mistreatment is not tolerated by providing appropriate training to all parties.”
	“Foster a supportive environment.”
	“Training programs should establish clear policies and create a safe reporting system, provide training for all staff and residents, encourage open communication, respond promptly and especially fairly to reports of mistreatment, and collaborate with other programs”
**Selection committees and processes need to acknowledge EDI**	“Blind file reviews for residency applicants.”
	“…ensure there are diverse voices on the hiring and selection panel.”
	“Training programs should standardize evaluation criteria (and make it transparent), provide implicit bias training (mandatory), and ensure diverse selection committees (representation)…”
	“Anonymous review of resident/faculty applications.”
	“Hiring committees should have undergone training to mitigate bias. The selection of the hiring committee should also reflect the diversity, equity, and inclusion we seek in our applicants. EDI should be a goal to strive for when selecting/ranking candidates.”

## Data Availability

The data presented in this study are available on request from the corresponding author. The data are not publicly available to protect patient privacy.

## Supplementary Materials

The following supporting information can be downloaded at: https://www.mdpi.com/article/10.3390/curroncol32110623/s1, Learner Survey.
