# Supplementary material for: Understanding Equity, Diversity, and Inclusion Within Canadian Radiation Oncology Training Programs: A National Survey of Residents and Fellows
Source: Curr Oncol. 2025 Nov 6;32(11):623. doi: 10.3390/curroncol32110623 (PMC12650942; doi:10.3390/curroncol32110623)
Supplement: Supplementary file 1 [file curroncol-32-00623-s001.zip › curroncol-3912943-supplementary.pdf]

Canadian Radiation Oncology Learning Environment Survey

Dear colleague,

You are being invited to participate in an anonymous research study because you are a current radiation oncology trainee in Canada. Our objective is to evaluate diversity within Radiation oncology in Canada, in order to inform opportunities to promote equity and inclusivity. Information is being collected on the learning and working environment which will hopefully be helpful to improving working conditions.

Your participation in the survey is voluntary. All data will be anonymized. If you do not wish to participate there will be no penalties for declining or withdrawing from the study and you will not be required to answer any questions you do not wish to.

Below is the link to the survey. Consent is implied by completing the survey which should take approximately 15 minutes to complete. After completing the survey, you will have the option to provide your email address in a separate link to enter a draw to receive a $20 gift card. Your email address will be kept separate from the survey responses.

Sincerely,

Researchers:
Dr. Jennifer Croke Department of Radiation Oncology University of Toronto
(416-946-4483)

Fellow/ Resident Investigators:
Dr. Stefan Allen – University of Toronto (902-489-3492)
Dr. Amanda Khan – University of Calgary (403-521-3642)

Study Team:
Dr. Shaun Loewen – Radiation oncologist, University of Calgary
Dr. Rachel Ellaway – Community Health Sciences, University of Calgary
Dr. Ian Gerard – Radiation Oncology, McGill University
Dr. Jolie Ringash – Radiation Oncology, University of Toronto
Dr. David Bowes – Radiation Oncology, Dalhousie University
Dr. Glen Bandiera – Temerty Faculty of Medicine, University of Toronto
Dr. Reshma Jagsi – Radiation Oncology, Emory School of Medicine

<u>Learner Survey</u>

Demographics

1. **Are you a Canadian Medical Graduate (i.e. obtained your MD degree in Canada) or an International Medical Graduate (MD obtained elsewhere, including the US)?**
   - Canadian medical graduate
   - International medical graduate

2. **Was/is your residency training at a Canadian institution?**
   - Yes
   - No

3. **What age (years) bracket do you fall within?**
   - 25-34
   - 35-44
   - 45-54
   - 55-64
   - 65-74
   - 75+

4. **What is your citizenship status?**
   - Born a Canadian citizen
   - Canadian Citizenship via Immigration
   - Permanent resident in Canada
   - Study / Work visa

5. **What is your marital status?**
   - Single
   - Divorced/separated
   - Widowed
   - Married/in a domestic relationship
   - I prefer not to answer this question

6. **Do you have any children (under the age of 18) or dependents you are responsible for or share care for?**
   - 0
   - 1
   - 2
   - 3
   - 4
   - 5
   - 6
   - 7

- 8
- 9

7. **What level of training are you currently in?**
- PGY 1
- PGY 2
- PGY 3
- PGY 4
- PGY 5
- Fellow
- Prefer Not to Answer

8. **What degree(s) have you earned? Please select all that apply.**
- MD or equivalent (eg: MBBS)
- Masters or equivalent
- PhD or equivalent
- JD
- MBA or equivalent

9. **Do any of your parents or guardians have a college/university degree?**
- One
- Both
- Neither
- Prefer not to answer

10. **Please indicate your approximate household income when you were a teenager**
- Less than $25,000
- $25,000 to $50,000
- $50,000 to $75,000
- $100,000 to $125,000
- $125,000 to $150,000
- $150,000 +
- I Prefer not to answer
- I don't know

11. **The 2016 Canadian census defined visible minorities as being a member of one of the following groups. Please select as many categories as you identify with.**
- South Asian (eg: Indian, Bangladeshi, Sri Lankan)
- Chinese
- Black
- Filipino
- Latin American
- Arab
- Southeast Asian (eg: Cambodian, Indonesian, Thai)

- West Asian (eg: Iranian)
- Korean
- Japanese
- First Nations
- Inuk (Inuit)
- Metis
- I prefer not to answer this question
- Other (please specify): [open text box]

12. **What is your primary first language**?
- English
- French
- Indigenous/ First nations language or dialect
- Another language
- I prefer not to answer
- (Please type in "NA" if not applicable)

13. What languages besides English do you currently know and would feel comfortable speaking in simple terms to patients?

14. What would you consider to be your religious or spiritual affiliation that you currently practice or associate with? Please select all that applies.
- Atheist / Agnostic / No religious or spiritual affiliation currently practiced
- Bahá'í Faith
- Buddhism
- Christianity (any, including Catholic, Protestant, Evangelical, etc.)
- Confucianism
- Hinduism
- Jainism
- Judaism
- Islam
- Native Spirituality
- Sikhism
- Spiritual
- I prefer not to answer
- Not listed. Please specify

15. From your name, physical appearance and/or anything you always wear, are you easily identifiable as a member of a specific religion?
- Definitely yes
- Probably yes
- Probably no
- Definitely no
- Not sure

16. What religion would people assume you belong to?

- Buddhism
- Christianity (any, including Catholic, Protestant, evangelical, etc.)
- Confucianism
- Hinduism
- Judaism
- Islam
- Native Spirituality
- Sikhism
- Other. Please specify:

17. Which of the following best describes your current gender identity/identities? (Select all that apply)

Tool Tips
[Agender] – Agender is a person who does not identify themselves as having a particular gender.
[Gender-fluid] -– Gender-fluid is a nonbinary gender identity that's not fixed and is capable of changing over time.
[Gender identity] – Gender identity means a person's internal sense of whether they are male, female, both or neither. It is a person's internal, deeply held sense of one's gender, and is not visible to others. Individuals may have more than one gender identity, and it can be fluid over time.
[Non-binary] -– Nonbinary gender is an umbrella term to describe any gender identity that does not fit into the gender binary of male and female. Nonbinary gender (also sometimes referred to as genderqueer) people may, for example, identify as having no gender, fall on a gender spectrum somewhere between male and female, or identify as totally outside binary gender identities.
[Transgender] -– Transgender refers to individuals whose gender identity or expression differs from societal expectations of the sex they were assigned at birth
[Two-spirit] -– Two-spirit is an umbrella term used to describe an Indigenous person who does not identify with colonial gender structures. It may be used to describe gender, sexual, and spiritual identity.

- Agender
- Gender-fluid
- Man
- Non-binary
- Transgender
- Two-spirit
- Woman
- I do not know
- I prefer not to answer
- Not listed, please specify

18. Which of the following best describes your sexual orientation(s)? (Select all that apply)

Tool Tips
[Asexual] – a spectrum that represents individuals who feel little to no sexual attraction
[Bisexual] – an individual attracted to more than one sex, gender, or gender identity
[Gay] – an individual who is emotionally, sexually, and/or romantically attracted to members of the same gender
[Lesbian] – a woman who is physically, sexually, and/or emotionally attracted to another woman or female-gendered person
[Queer] – an umbrella term for the whole non-heterosexual community; queerness intentionally has no single definition beyond "not straight"
[Straight] – an individual who is emotionally, sexually, and/or romantically attracted to members of the opposite gender
[Two-spirit] – an umbrella term used to describe an Indigenous person who does not identify with colonial gender structures. It may be used to describe gender, sexual, and spiritual identity
[Pan-sexual] – sexual, romantic, or emotional attraction towards people regardless of their sex or gender identity

- Asexual
- Bisexual
- Gay
- Lesbian
- Queer
- Straight / Heterosexual
- Two-spirit
- Pansexual
- I self-identify as…[free form text field]
- I do not know / Questioning
- I prefer not to answer

19. **Do you identify as a person with a disability?** The *Accessible Canada Act* defines disability as "any impairment, including a physical, mental, intellectual, cognitive, learning, communication or sensory impairment - or a functional limitation - whether permanent, temporary or episodic in nature, or evident or not, that, in interaction with a barrier, hinders a person's full and equal participation in society."

- Yes
- No
- I prefer not to answer

20. If so, what do you regard as your disability? (Select all that apply)

- Blind/visual impairment
- Deaf/hearing impairment
- Speech/communication disability
- Mental health disability
- Mobility/physical disability

- Emotional disability
- Cognitive disability
- A health condition that affects your ability to be a physician
- Not applicable
- I prefer not to answer this question
- I prefer to self-describe, please specify below [open text box]

Job Perceptions

21. All in all I am satisfied with my program

- Strongly disagree
- Disagree
- Neither agree nor disagree
- Agree
- Strongly agree
- Any response will link to Text Box., please elaborate

22. **How often have you thought about moving to a different training program?**

- Never
- Once or twice
- Sometimes
- Often
- Many times
- Any response will link to Text Box., please elaborate

23. **How often have you felt regret about deciding to become a physician?**

- Never
- Once or twice
- Sometimes
- Often
- Many times
- Any positive response will link to Text Box., please elaborate

24. Thinking about the past academic year, how would you rate the <u>culture of respect</u> in your residency program? *Culture of respect refers to the attitudes, behaviors, and standards of your colleagues as related to access to, inclusion of, and level of respect for individual and group needs, abilities, and potential across the spectrum of diverse backgrounds and identities.*

- Excellent
- Very Good
- Good
- Adequate
- Poor
- Very Poor

Mentorship

25. A formal mentorship program exists within my residency training program

- Yes
- No

26. How much do you turn to peers (close to my level colleagues) for "peer mentorship?"

- Never
- Less than Monthly
- Monthly
- Weekly
- Daily

27. I currently have at least one faculty mentor

- Yes
- No

If yes, what do you discuss with your mentor, select all that are applicable

- Clinical work
- Research
- Networking
- Work-life balance
- Career planning
- Fellowship
- Leadership opportunities
- Studying techniques
- Teaching opportunities
- Not applicable
- Not listed, please specify

28. It is important I have a mentor with similar demographic characteristics to me

- Strongly disagree
- Disagree
- Neither agree nor disagree
- Agree
- Strongly agree

29. How easy has it been for you to identify someone whose career could serve as a model for your own?

- Very easy
- Easy
- Neither easy or difficult
- Difficulty
- Very difficult

30. **Overall, I am happy with the mentorship I currently receive**
- Very satisfied
- Somewhat satisfied
- Neither satisfied nor dissatisfied
- Somewhat dissatisfied
- Very dissatisfied

31. **People like myself are under-represented in my residency program**
- Yes
- No
- [IF YES, please elaborate]

32. **There are equity and diversity biases/obstacles for entry in my Radiation Oncology Residency Training Program**
- Yes
- No
- [IF YES, please elaborate]

Academics:

33. **How many peer-reviewed publications have you been an author on?**
- 0
- <5
- 5-10
- 10-25
- 25-50
- 50-100
- >100

34. **I desire a career with a strong research component**
- Strongly disagree
- Disagree
- Neither agree nor disagree
- Agree
- Strongly agree

35. **I desire a career with a strong education component**
- Strongly disagree
- Disagree
- Neither agree nor disagree
- Agree
- Strongly agree

36. I am pursuing a additional postgraduate degree during my residency (e.g. Master or PhD) during my residency

- Yes
- No

Harassment

37. During your residency/ fellowship program have you ever felt discriminated against because of any of the following? Check all that apply. *Discrimination is defined as unjust or prejudicial treatment based on the grounds of race, age, gender, sex, and/or other traits or characteristics. Discrimination includes but it is not limited to examples such as inequity in work assignments, evaluations/assessments, distribution of resources/support, compensation, and hiring practices. Discrimination can be direct or indirect, subtle or overt.*

- Gender
- Gender identity
- Age
- Race/ethnicity
- Sexual orientation
- National origin
- Disability
- Religion
- Marital status
- Socioeconomic status
- Childcare responsibilities, other caretaking responsibilities
- Level of education (Master vs Doctoral)
- Political view
- Not listed. Please elaborate:

38. **How often, if at all, did you personally experience discrimination during your residency/ fellowship program?**

Discrimination is defined as unjust or prejudicial treatment based on the grounds of race, age, gender, sex, and/or other traits or characteristics. Discrimination includes but it is not limited to examples such as inequity in work assignments, evaluations/assessments, distribution of resources/support, compensation, and hiring practices. Discrimination can be direct or indirect, subtle or overt.

- Never
- Once
- 2 – 4 times
- 5 – 10 times
- Regularly / On an ongoing basis

39. Since the start of your training program, have you encountered harassment at your workplace. Please select all that apply. *Harassment refers to a course of vexatious comment or conduct which the person knows or ought reasonably to know is unwelcome.*

- Yes, happened to me
- Yes, I witnessed it happening to someone else
- Yes, I heard about it happening to someone else
- No

40. **How often did you personally experience harassment during the course of your training program? (If above question indicated "happened to me")**

- Once
- 2 – 4 times
- 5 – 10 times
- Regularly / On an ongoing basis

41. Please rate your level of agreement with the following statement. I feel comfortable reported harassment incidents at my workplace.

- Strongly disagree
- Disagree
- Neither agree nor disagree
- Agree
- Strongly agree

42. What was the role of the person(s) who harassed / discriminated against you? Check all that apply.

- Faculty member
- Nurse
- Other allied health professional
- Resident / Clinical Fellow
- MD student / Other learner
- Lab worker
- Staff (administrative, non-faculty)
- Patient / Patient family
- Not listed. Please specify the role, but do not provide a name.

43. Was the person who harassed / discriminated against you someone in a position to directly affect your academic, and/or professional opportunities?

- Yes
- No
- Not sure
- Does not apply / prefer not to say

44. **Did you tell anyone about these experiences**?

- Yes
- No
- If no, please elaborate

45. If you experienced harassment/ discrimination perpetrated by a patient/family members, were you immediately supported by your supervisor?

- Yes
- No- they were not available
- No- I did not think they would be supportive
- No- for other reasons
- Does not apply

46. If you experienced harassment/ discrimination perpetrated by a patient/ family member, did you seek support from your residency training program?

- Yes
- No
- Does not apply
- [IF YES, please elaborate]
- [IF NO, please elaborate]

47. If you experienced harassment/ discrimination perpetrated by a patient/family members, was it based on the following (select all that apply)

- Gender
- Age
- Race/ethnicity
- Sexual orientation
- National origin
- Disability
- Religion
- Other
- Does not apply

48. Within your training program/university, is there training provided to address sexual harassment?

- Yes
- No
- Do not know

49. Within your training program/university, is there training provided to address anti-racism?

- Yes
- No
- Do not know

50. **Within your training program/ university, is there training provided to address LGBTQ health?**

- Yes
- No

- Do not know

51. **Within your residency program/university, is there training provided to address other forms of discrimination?**
    - Yes
    - No
    - Do not know

52. **What format is the training for sexual harassment?**
    - Orientation when starting the job
    - Annual online learning
    - Occasional videos
    - Occasional in-person presentations
    - Reading revised policies
    - Not listed. Please elaborate (Text Box)

53. **What format is the training for anti-racism?**
    - Orientation when starting the job
    - Annual online learning
    - Occasional videos
    - Occasional in-person presentations
    - Reading revised policies
    - Not listed. Please elaborate (Text Box)

54. **What format is the training for other forms of discrimination?**
    - Orientation when starting the job
    - Annual online learning
    - Occasional videos
    - Occasional in-person presentations
    - Reading revised policies
    - Not listed. Please elaborate

55. **What format is the training for LGBTQ education?**
    - Orientation when starting the job
    - Annual online learning
    - Occasional videos
    - Occasional in-person presentations
    - Reading revised policies
    - Not listed. Please elaborate

56. **What should training programs do to address learner mistreatment?**

57. **What should training programs do to advance equity diversity and inclusion in the**

workplace?

58. What should training programs do to make resident selection or faculty hiring practices more equitable?

59. **Please rate the climate of your primary department (or division, if more relevant) on the following continuum by circling a number:** *Climate refers to the attitudes, behaviors, and standards of your colleagues as related to access to, inclusion of, and level of respect for individual and group needs, abilities, and potential across the spectrum of diverse backgrounds and identities.*

Racist	1	2	3	4	5	Non-racist
Homogeneous	1	2	3	4	5	Diverse
Non-sexist	1	2	3	4	5	Sexist
Collaborative	1	2	3	4	5	Individualistic
Cooperative	1	2	3	4	5	Competitive
Homophobic	1	2	3	4	5	Non-homophobic
Not supportive	1	2	3	4	5	Supportive

VERSION FRANÇAISE

Cher collègue,

Vous êtes invité à participer à une étude de recherche anonyme parce que vous êtes actuellement un médecin résident ou un médecin en radio-oncologie au Canada. Cette étude vise à comprendre la diversité des données démographiques de l'effectif de médecins radio-oncologues canadiens, afin de promouvoir l'équité et l'inclusion au sein de la profession. Des informations seront recueillies concernant l'environnement d'apprentissage et le milieu de travail. L'objectif de la présente étude est de pouvoir utiliser les informations recueillies afin d'améliorer les conditions du milieu de travail. Cette étude a été approuvée par les comités d'éthique de la recherche de l'Université de Calgary et du Réseau universitaire de santé.

Votre participation au sondage est volontaire. Toutes les données seront rendues anonymes. Si vous préférez ne pas participer à l'étude, il n'aura aucune conséquence en raison de votre refus. De surcroît, votre retrait sera permis en tout temps. Vous ne serez pas obligé de répondre aux questions auxquelles vous ne souhaitez pas répondre.

Vous trouverez ci-dessous le lien menant vers le sondage. Le consentement à la participation est implicite en raison de l'exécution du sondage, ce qui devrait nécessiter environ 15 minutes. Après avoir rempli le sondage, vous aurez l'option de fournir votre adresse électronique. En ce faisant, vous courrez la chance de gagner une carte-cadeau au montant de 20$. Votre adresse électronique sera fournie à travers un site web distinct de celle de l'étude et conservée séparément des réponses au sondage.

Cordialement,

Dr. Shaun Loewan
Radio-oncologue
Tom Baker Cancer Centre
shaun.loewen@albertahealthservices.ca

Dre. Jennifer Croke
Radio-oncologue
Princess Margaret Cancer Centre
jennifer.croke@rmp.uhn.ca

<u>Sondage pour résidents</u>

Données démographiques

1. Êtes-vous titulaire d'un diplôme canadien en médecine (c'est-à-dire que vous avez obtenu votre diplôme en médecine au Canada) ou titulaire d'un diplôme en médecine obtenu à l'extérieur de Canada (diplôme de médecine obtenu ailleurs, incluant aux États-Unis) ?
   a. Diplômé canadien en médecine
   b. Diplômé international en médecine

2. **Est-ce que votre formation de résidence en radio-oncologie a été/est effectuée dans un établissement canadien?**
   a. Oui
   b. Non

3. **Dans quelle tranche d'âge vous situez-vous?**
   - 25-34
   - 35-44
   - 45-54
   - 55-64
   - 65-74
   - 75+

4. **Quel est votre statut de citoyenneté canadienne?**
   - Né(e) une citoyen(ne) canadien(ne)
   - Citoyen(ne) canadien(ne) naturalisé(e)
   - Résident(e) permanent(e) au Canada
   - Visa d'étude/de travail
   - Je préfère ne pas répondre

5. **Quel est votre état civil?**
   - Célibataire
   - Séparé(e)/divorcé(e)
   - Veuf/veuve
   - Marié(e)/conjoint(e) de fait
   - Je préfère ne pas répondre

6. **Avez-vous des enfants (âgés de moins de 18 ans) ou des personnes à charge desquelles vous vous occupez, soit à temps plein ou en garde partagée?**
   - 0
   - 1
   - 2

- 3
- 4
- 5
- 6
- 7
- 8
- 9

7. **Quel niveau de formation complétez-vous actuellement ?**
    - R1
    - R2
    - R3
    - R4
    - R5
    - Fellow clinique
    - Je préfère ne pas répondre

8. **Quel(s) diplôme(s) détenez-vous? Veuillez choisir tous ceux qui s'appliquent.**
    - Médecine ou équivalent
    - Maitrise ou équivalent
    - Doctorat ou équivalent
    - Doctorat en droit (*juris doctor*) ou équivalent
    - Magistère en gestion (*MBA*) ou équivalent

9. **Vos parents ou gardiens sont-ils/elles des titulaires d'un diplôme universitaire?**
    - Un(e)
    - Les deux
    - Ni l'un(e) ni l'autre
    - Je préfère ne pas répondre

10. **Indiquez laquelle de ces catégories correspond le mieux à votre revenu familial annuel lorsque vous étiez adolescent?**
    - Moins de 25 000$
    - 35 000$ à 75 000$
    - 75 000$ à 100 000$
    - 100 000$ à 125 000$
    - 125 000$ à 150 000$
    - Plus de 150 000$
    - Je préfère ne pas répondre
    - Je ne sais pas

11. **Le Recensement canadien de 2016 définit les minorités visibles comme étant constituées de plusieurs groupes, tel qu'énuméré ci-dessous. Veuillez s'il vous plaît sélectionner le(s) catégorie(s) avec laquelle ou lesquelles vous identifiez.**

- Sud-asiatique (ex : indien, bangladais, sri lankais)
- Chinois(e)
- Noir(e)
- Philippin(ne)
- Latino/Latina-américaine
- Arabe
- Sud-est asiatique (ex : cambodgien, indonésien, thaïlandais)
- Ouest-asiatique (ex: iranien)
- Coréen(ne)
- Japonais(e)
- Premières nations
- Inuk (Inuit)
- Métis
- Je préfère ne pas répondre
- Autre [champ de saisie de texte libre]

12. Quel est votre langue maternelle?

- Anglais
- Français
- Langue ou dialecte autochtone/Premières nations
- Autre langue
- Je préfère ne pas répondre

(Veuillez écrire « NA » si sans objet)

13. **Quelle(s) langue(s) connaissez-vous présentement, mis à part le français (et l'anglais, si applicable)? Une connaissance suffisante nécessite la capacité de s'entretenir avec des patients dans ladite ou lesdites langue(s).**

14. **Quelle est votre appartenance religieuse ou spirituelle présentement?** Veuillez s'il vous plaît sélectionner le(s) catégorie(s) avec laquelle ou lesquelles vous identifiez.

- Agnostique, athée ou aucune appartenance religieuse ou spirituelle
- Bouddhiste
- Chrétien(ne) (incluant catholique, protestant(e), évangélique ou autre)
- Confucianisme
- Hindouisme
- Jaïnisme
- Judaïsme
- Islam
- La spiritualité autochtone
- Sikhisme
- Spirituel
- Je préfère ne pas répondre
- Ne figure pas sur la liste ci-dessus, veuillez préciser.

15. **Est-ce que vous pourrez être identifiés comme appartenant à une religion précise en raison de votre nom, votre apparence physique et/ou un symbole ou un item que vous portez quotidiennement?**
    - Oui (sans équivoque)
    - Oui (probablement)
    - Non (probablement pas)
    - Non (sans équivoque)
    - Pas certain(e)

16. **À quelle religion est-ce qu'on vous associe?**

    - Bouddhiste
    - Chrétien(ne) (incluant catholique, protestant(e), évangélique ou autre)
    - Confucianisme
    - Hindouisme
    - Judaïsme
    - Islam
    - La spiritualité autochtone
    - Sikhisme
    - Autre, veuillez préciser.

17. **Lequel des éléments suivants décrit le mieux vos/votre identité(s) de genre(s) ? (Choisir toutes les réponses applicables)**

    Info-bulles

    [Agenre] – Une personne qui ne se reconnait dans aucune identité de genre.
    [Genre fluide] - Une identité de genre non-binaire qui est fluide, les personnes pouvant se définir d'une façon ou d'une autre à différents moments.
    [Identité de genre] – L'identité de genre d'une personne est la catégorie de genre à laquelle elle estime appartenir (soit homme, femme, les deux ou aucun). C'est une croyance profondément personnelle qui n'est pas visible aux autres. Des personnes peuvent posséder plus qu'une identité de genre et celle-ci pourrait varier au cours du temps.
    [Non binaire] - Terme générique qui décrit une identité de genre qui ne relève pas du binaire de genre homme-femme. Par exemple, une personne de genre non binaire (souvent synonyme de genderqueer*) peut s'identifier comme n'ayant aucun genre ou ne se considérer pas comme étant exclusivement femme ou exclusivement homme.*
    [Transgenre] – Une personne ayant une identité de genre ou une expression d'identité de genre différente des attentes de la société associées au genre qui leur a été assigné à la naissance.

[Bispirituel(le)] - Terme générique utilisé par des autochtones nord-américains pour désigner des personnes non-conformes aux structures postcoloniales de genre, de sexualité et/ou de l'identité spirituelle.

- Agenre
- Genre fluide
- Homme
- Non binaire
- Transgenre
- Bispirituel(le)
- Femme
- Je ne sais pais
- Je préfère ne pas répondre
- Autre, veuillez préciser

18. Lequel des éléments suivants décrit le mieux vos/votre orientation(s) sexuelle(s) ? (Choisir toutes les réponses applicables)

- Asexué
- Bisexuel(le)
- Gay
- Lesbienne
- *Queer*
- Hétérosexuel(le)
- Bispirituel(le)
- Pansexuel(le)
- Je m'identifie comme… *[champ de saisie de texte libre]*
- Je ne sais pais/remis en question
- Je préfère ne pas répondre

Info-bulles
[Asexué] - Un éventail de personnes qui ne ressentent pas ou peu d'attirance sexuelle.
[Bisexuel(le)] - Une personne qui éprouve de l'attirance sexuelle ou des sentiments amoureux pour plus d'un sexe, d'un genre ou d'une identité de genre.
[Gay] – Une personne qui éprouve de l'attirance sexuelle ou des sentiments amoureux pour des membres du même genre.
[Lesbienne] – Une femme qui éprouve de l'attirance sexuelle ou des sentiments amoureux pour une autre femme ou une personne s'auto-identifiant comme une femme.
[Queer] – Terme générique pour la communauté non-hétérosexuelle dans son ensemble; le mot queerness *est expressément ambigüe et ne comporte aucune définition précise, mis à part la non-hétérosexualité.*

[Hétérosexuel(le)] – Une personne qui éprouve de l'attirance sexuelle et/ou des sentiments amoureux pour des membres du genre opposé.
[Bispirituel(le)] - Terme générique utilisé par des autochtones nord-américains pour désigner les personnes non-conformes aux structures postcoloniales de genre, de sexualité et/ou de l'identité spirituelle.
[Pansexuel(le)] – Se dit d'une personne qui est attirée sexuellement ou émotionnellement par une autre sans égard au genre ou au sexe de cette dernière.

19. **Vous considérez-vous comme ayant un handicap, tel que défini par la *Loi canadienne sur l'accessibilité*, soit une « déficience notamment physique, intellectuelle, cognitive, mentale ou sensorielle, trouble d'apprentissage ou de la communication ou limitation fonctionnelle, de nature permanente, temporaire ou épisodique, manifeste ou non et dont l'interaction avec un obstacle nuit à la participation pleine et égale d'une personne dans la société » ?**
    - Oui
    - Non
    - Je préfère ne pas répondre

20. **Si oui, qu'est-ce que vous considérez comme étant votre handicap? (Sélectionnez toutes les réponses qui s'appliquent)**
    - Aveugle/handicap visuel
    - Sourd/trouble auditif
    - Troubles de la parole
    - Problèmes de santé mentale
    - Mobilité/handicap physique
    - Troubles émotionnels
    - Handicap cognitif
    - État de santé qui influence votre capacité d'agir comme médecin
    - Sans objet
    - Je préfère ne pas répondre
    - Je préfère m'auto-décrire, veuillez préciser ci-dessous [champ de saisie de texte libre]

Perception du domaine de travail

21. **Dans l'ensemble, je suis satisfaite de mon programme de formation**
    - en désaccord
    - ni d'accord, ni en désaccord
    - d'accord
    - tout à fait d'accord
    - Toute réponse mènera à un champ de saisie de texte libre, veuillez fournir des précisions.

22. Avez-vous envisagé un transfert à un autre programme?

- Jamais
- Une ou deux fois
- Parfois
- Souvent
- Plusieurs fois
- Toute réponse mènera à un champ de saisie de texte libre, veuillez fournir des précisions.

23. Est-ce que vous avez éprouvé du regret en lien avec votre choix de devenir médecin?

- Jamais
- Une ou deux fois
- Parfois
- Souvent
- Plusieurs fois
- Toute réponse positive mènera à un champ de saisie de texte libre, Veuillez fournir des précisions.

24. **Comment évalueriez-vous la culture de respect au sein de votre département au cours de l'année écoulée?** La culture de respect au travail reflète un milieu de travail qui valorise et respecte tous les employés, peu importe leur situation personnelle, c'est-à-dire leur origine ethnique, leur sexe, leurs capacités ou leurs incapacités, leur âge, leurs caractéristiques physiques, leur religion, les valeurs de leur culture, leurs responsabilités relatives au foyer ou à la famille, et leur orientation sexuelle.

- Excellent
- Très bon
- Bon
- Satisfaisant
- Insuffisant
- Très insuffisant

Mentorat

25. Un programme structuré de mentorat existe au sein de mon programme de formation.

- Oui
- Non

26. À quelle fréquence sollicitez-vous du mentorat parmi vos pairs?

- Jamais
- Moins d'une fois par mois
- Sur une base mensuelle
- Sur une base hebdomadaire

- Sur une base quotidienne

27. J'ai présentement au moins un(e) mentor(e) qui son personnel(le) de la faculté

- Oui
- Non

Si oui, quel sujets abordez-vous avec votre mentor(e)? **(Sélectionnez toutes les réponses qui s'appliquent)**

- Travail clinique
- Recherche
- Réseautage
- Conciliation travail-vie personnelle
- Planification de carrière
- Fellowship clinique
- Possibilités de gestion/leadership
- Techniques d'études
- Possibilités pédagogiques
- Sans objet
- Autre, veuillez préciser

28. C'est important que mon mentor présente des caractéristiques démographiques semblables aux miennes.

- pas du tout d'accord
- en désaccord
- ni d'accord, ni en désaccord
- d'accord
- tout à fait d'accord

29. Est-ce que c'est facile pour vous d'identifier quelqu'un dont la carrière pourrait servir de modèle?

- Très facile
- Facile
- Pas facile, ni difficile
- Difficile
- Très difficile

30. Dans l'ensemble, je suis content(e) de mon expérience de mentorat

- Très satisfait(e)
- Quelque peu satisfait(e)
- Neutre
- Assez insatisfait(e)
- Très insatisfait(e)

31. Des gens comme moi sont sous-représenté(e)s dans mon programme de formation.
- Oui
- Non

[Si oui, veuillez fournir des précisions.]

32. Il y a des entraves à l'admission à mon programme de formation, et ce, au niveau de l'équité et la diversité.
- Oui
- Non

[Si oui, veuillez fournir des précisions.]

Universitaires

33. Combien de publications évaluées par les pairs avez-vous publié (à titre d'auteur, soit principal ou autre)?
- 0
- <5
- 5-10
- 10-25
- 25-50
- 50-100
- >100

34. Je désire entamer une carrière avec une forte composante en recherche.
- pas du tout d'accord
- en désaccord
- ni d'accord, ni en désaccord
- d'accord
- tout à fait d'accord

35. **Je désire entamer une carrière avec une forte composante pédagogique.**
- pas du tout d'accord
- en désaccord
- ni d'accord, ni en désaccord
- d'accord
- tout à fait d'accord

36. Je poursuis mes études de troisième cycle (*postgraduate degree*, ex : maîtrise ou doctorat) pendant ma formation en radio-oncologie.
- Oui
- Non

Harcèlement

37. Avez-vous l'impression que vous fassiez l'objet de **discriminations** lors de votre formation médicale, de manière générale? Si oui, veuillez sélectionner toutes les raisons qui s'appliquent. La discrimination est un traitement injuste ou préjudiciable envers une personne ou un groupe de personnes (en raison de leur race, âge, genre, sexe ou autre caractéristiques) qui les empêche d'avoir pleinement accès aux occasions et aux avantages auxquels ont accès d'autres membres de la société. La discrimination comprend donc notamment, mais pas exclusivement, des affectations inéquitables de travail, ainsi que du traitement inéquitable affectant des processus d'évaluation, l'allocation de ressources, l'indemnisation et les pratiques d'embauches. La discrimination peut être directe ou indirecte, ainsi que déguisée.
    - Genre
    - Identité de genre
    - Âge
    - Race ou origine ethnique
    - Orientation sexuelle
    - Origine nationale
    - Handicap
    - Religion
    - État civil
    - Statut socioéconomique
    - Responsabilités liées à la garde des enfants ou autres personnes à charge
    - Niveau de scolarité (maîtrise versus doctorat)
    - Opinion politique
    - Autre, veuillez préciser

38. Si applicable, combien de fois avez-vous fait l'objet de discriminations lors de votre formation médicale?
    - Jamais
    - Une fois
    - 2 à 4 fois
    - 5 à 10 fois
    - Sur une base régulière

39. **Depuis le début de votre programme de formation, avez-vous subi du harcèlement? Si oui, veuillez sélectionner toutes les réponses qui s'appliquent. Le harcèlement se définit comme un comportement inopportun et injurieux envers une ou d'autres personnes, et dont l'auteur savait ou aurait raisonnablement dû savoir qu'il pouvait offenser ou causer un préjudice**.
    - Oui, il m'est arrivé
    - Oui, j'étais témoin du harcèlement envers d'autres
    - Oui, j'ai entendu dire que d'autres personnes ont fait l'objet du harcèlement
    - Non

40. Combien de fois avez-vous personnellement fait l'objet du harcèlement durant votre programme de formation? (Si la réponse à la question ci-dessus est « Oui, il m'est arrivé)

- Jamais
- Une fois
- 2 à 4 fois
- 5 à 10 fois
- Sur une base régulière

41. Indiquez dans quelle mesure vous êtes d'accord avec l'énoncé suivant : « Je me sens à l'aise de dénoncer du harcèlement dans mon milieu de travail. »

- pas du tout d'accord
- en désaccord
- ni d'accord, ni en désaccord
- d'accord
- tout à fait d'accord

42. Quel était le rôle de la personne de qui le harcèlement ou les discriminations ont émanés? Veuillez sélectionner toutes les réponses qui s'appliquent.

- Personnel de la faculté
- Infirmière
- Autre professionnel de la santé
- Résident(e) ou fellow clinique
- Étudiant(e) en médicine ou autre étudiant(e)
- Personnel de recherche
- Personnel administrative ou de gestion
- Patient(e) ou famille d'un(e) patient(e)
- Autre. Veuillez préciser leur rôle, sans toutefois fournir leur nom.

43. Est-ce que la personne qui vous a harcelé ou qui a commis des actes discriminatoires envers vous pourrait avoir un impact direct sur vos possibilités de carrières et/ou pédagogiques?

- Oui
- Non
- Je ne sais pas
- Je préfère ne pas répondre / Sans objet

44. Avez-vous partagé votre expérience avec d'autres?

- Oui
- Non,

- Si non, veuillez fournir des précisions.

45. Si vous avez fait l'objet du harcèlement ou des discriminations par un patient et/ou sa famille, avez-vous bénéficié du soutien de votre supérieur sans délai?

- Oui
- Non – il/elle n'était pas disponible
- Non – je ne pensais pas qu'il/elle m'appuyerait
- Non – autre raison
- Sans objet

46. Si vous avez fait l'objet du harcèlement ou des discriminations par un patient et/ou sa famille, avez-vous cherché à obtenir de l'aide de votre programme de formation?

- Oui
- Non
- Sans objet
- Si oui, veuillez préciser.
- Si non, veuillez préciser.

47. Si vous avez fait l'objet du harcèlement ou des discriminations par un patient et/ou sa famille, c'était pour quelle raison? Veuillez sélectionner toutes les réponses qui s'appliquent.

- Genre
- Âge
- Race ou origine ethnique
- Orientation sexuelle
- Origine nationale
- Handicap
- Religion
- Autre
- Sans objet

48. Est-ce que votre programme de formation offre de l'enseignement au sujet du harcèlement sexuel?

- Oui
- Non
- Je ne sais pas

49. Est-ce que votre programme de formation offre de l'enseignement afin de lutter contre le racisme?

- Oui
- Non
- Je ne sais pas

50. Est-ce que votre programme de formation offre de l'enseignement axé sur la santé LGBTQ?
- Oui
- Non
- Je ne sais pas

51. Est-ce que votre programme de formation offre de l'enseignement au sujet d'autres types de discriminations?
- Oui
- Non
- Je ne sais pas

52. Quelle formule de la formation est dispensée pour le harcèlement sexuel?
- Programme d'orientation (quand on commence l'emploi)
- Apprentissage annuel sur l'internet
- Vidéos ponctuels
- Présentations ponctuels en présentiel
- Lecture des versions révisées des politiques écrites
- Autre, veuillez préciser [champ de saisie de texte libre]

53. Quelle formule de la formation est dispensée pour la lutte contre le racisme?
- Programme d'orientation (quand on commence l'emploi)
- Apprentissage annuel sur Internet
- Vidéos ponctuels
- Présentations ponctuels en présentiel
- Lecture des versions révisées des politiques écrites
- Autre, veuillez préciser [champ de saisie de texte libre]

54. Quelle formule de la formation est dispensée pour d'autres formes de discrimination?
- Programme d'orientation (quand on commence l'emploi)
- Apprentissage annuel sur Internet
- Vidéos ponctuels
- Présentations ponctuels en présentiel
- Lecture des versions révisées des politiques écrites
- Autre, veuillez préciser [champ de saisie de texte libre]

55. Quelle formule de la formation est dispensée pour l'éducation LBGTQ?
- Programme d'orientation (quand on commence l'emploi)
- Apprentissage annuel sur Internet
- Vidéos ponctuels
- Présentations ponctuels en présentiel
- Lecture des versions révisées des politiques écrites
- Autre, veuillez préciser [champ de saisie de texte libre]

56. Comment est-ce que les programmes de formation peuvent s'attaquer au problème du mauvais traitement des stagiaires?

57. Comment est-ce que les programmes de formation peuvent favoriser l'équité, la diversité et l'inclusion dans les milieux professionnels?

58. Comment est-ce que les programmes de formation peuvent favoriser l'équité dans les processus d'embauche pour les médecins résidents ainsi que la faculté?

59. **Veuillez évaluer le climat dans votre département principal (ou votre division, si applicable). Utilisez l'échelle ci-dessous et encerclez le chiffre correspondant à votre évaluation.** Le climat reflète les attitudes, les comportements et les standards de vos collègues en fonction de l'accès et de l'inclusion, ainsi que du respect, des besoins particuliers des individus ou des groupes ayant des identités, des capacités, des cultures, des compétences, des expériences et des points de vue diverses.

Raciste	1	2	3	4	5	Non-raciste
Homogène	1	2	3	4	5	Diversifié
Non-sexiste	1	2	3	4	5	Sexiste
Collaboratif	1	2	3	4	5	Individualiste
Coopératif	1	2	3	4	5	Compétitif
Homophobe	1	2	3	4	5	Non-homophobe
Environnement défavorable	1	2	3	4	5	Environnement favorable
